# Decision regret following surgical management of pilonidal disease

**DOI:** 10.1111/codi.17152

**Published:** 2024-09-25

**Authors:** J. Banks, E. Lee, M. J. Lee, S. R. Brown, PITSTOP Management Group, K. Ali, R. Brady, G. Branagan, S. Chaudri, F. Di Fabio, G. Dennison, D. Donnelly, M. Evans, F. Gerald, S. Gonzalez, J. Grainger, A. Hardy, N. Husain, S. Kapur, K. Keogh, M. Lim, P. Mackey, Y. Maeda, S. Mangam, F. Mazarelo, K. Muhammad, N. Pawa, L. Pearce, J. Pitt, R. Rajaganeshan, P. Shackley, R. Simmonds, R. Stevenson, J. Torkington, P. Vaughan‐Shaw, Vimalachandran Dale, J. Wilson

**Affiliations:** ^1^ Division of Clinical Medicine, Faculty of Medicine, Dentistry & Health University of Sheffield Sheffield UK; ^2^ Sheffield Clinical Trials Research Unit, School of Health and Related Research (ScHARR) University of Sheffield Sheffield UK; ^3^ Institute for Applied Health Research, College of Medical and Dental Sciences, University of Birmingham Birmingham UK

**Keywords:** Pilonidal Disease, Proctology, Surgery

## Abstract

**Aim:**

Surgical decision making in the context of pilonidal disease (PD) can be challenging. Current evidence for the management of PD is inadequate and optimum treatment is not clear. This paper reports on patient experience of shared decision making (SDM) and decision regret following surgical management of PD.

**Method:**

The Pilonidal Trial. Studying the Treatment Options (PITSTOP) study (ISRCTN95551898) is a prospective cohort study of patients with PD treated between May 2019 and March 2022. This subanalysis reports the results of quantitative data capture between baseline and 6 months post‐procedure. Baseline data consisted of patient and disease characteristics, surgical procedure and impression of SDM. Post‐procedure data consisted of operative outcomes and decision regret. Multiple linear regression analysis was used to analyse the relationship between clinical outcomes and decision regret.

**Results:**

Overall, 677 patients were included, and follow‐up data to 6 months were available for 476 (71%). Most (59.5%) patients underwent major excisional surgery; 45.1% of patients experienced a postoperative complication. Participant impression of SDM was positive, with a median CollaboRATE mean‐score response of 3 (interquartile range: 3–4). Of the patients who underwent a ‘leave open’ approach, 20.6% were dissatisfied or very dissatisfied with their treatment. Postoperative complications (*β* = 3.21, 95% CI: −12.75 to 7.25, *p* < 0.001) and disease recurrence (*β* = 11.5, 95% CI: −10.6 to 9.4, *p* < 0.001) were both associated with higher rates of decision regret.

**Conclusion:**

The clinical outcomes, postoperative complications and recurrence, were associated with higher levels of decision regret. Surgeons treating patients with PD should practice SDM and ensure that patient priorities inform treatment approach.

**What does this paper add to the literature?**

Surgical decision making is a complex process involving both patient and surgeon; it requires careful discussion of the risk and benefits of potential procedures and treatment options. This study shows patient experience of shared decision making. It highlights that complications and disease recurrence drive patient regret over procedures.

## INTRODUCTION

Pilonidal disease (PD) is a common clinical condition affecting around 26 per 100,000 people; the highest incidence is reported in young men patients of working age, in whom up to 8.8% are affected [1, 2]. In clinical practice, the presentation of PD can vary greatly. It most commonly affects the sacrococcygeal area and is characterized by ingrowing or implanted hairs in the natal cleft. The extent of the disease varies between individuals; consequently, disease patterns range from simple sinuses with minimal pain and discharge to complex abscess cavities and acute infection. The disease can have a significant impact on the personal, social and economic activities of affected individuals [3].

There is currently no clear evidence for the optimal surgical management of chronic PD, and this is reflected in the wide variety of practice reported among UK surgeons [4]. In the elective setting, procedures often involve excision of the sinus with or without primary closure. This approach leaves patient with large wounds, resulting in prolonged healing times and significant disruption to work and social life [5]. Minimally invasive procedures, such as phenol injection or fistuloscopy/diathermy, are increasingly being used for minimally symptomatic PD. However, uptake of minimally invasive procedures has not been universal and there are some concerns surrounding higher recurrence rates with these techniques [6]. As PD is most commonly a disease of young, healthy and economically active individuals, it is important that treatment options are associated with fast healing times and minimal complications to reduce the personal and economic burden of this disease.

Surgical decision making is a complex process involving both patient and surgeon; it requires careful discussion of the risk and benefits of potential procedures and treatment options, which in the context of PD can be particularly challenging. The optimum treatment for PD, namely one that is both easy to perform and results in rapid healing with minimal complications, is not clear. Current evidence for the management of PD is inadequate as it is largely based on case series or nonrandomized comparative trials; moreover, where randomized control trials have been carried out, over 90% are single‐centre studies, limiting the generalizability of the results [7]. Consequently, clinicians may advocate for procedures that have a limited evidence basis and patients may not be informed of the range of treatment options available for PD [7]. Several studies have demonstrated that shared decision making improves patient satisfaction and reduces decision regret [8]; however, little work has been carried out on shared decision making in the context of PD.

The Pilonidal Trial. Studying the Treatment Options (PITSTOP) study was designed to address the lack of robust research in the management of PD following a National Institute for Health Research (NIHR) call to assess the different treatment options in PD and to identify both treatment and outcome priorities for patients with PD [9]. This paper reports on patient's experience of shared decision making and decision regret following surgical management of PD.

## METHOD

The PITSTOP study was a prospective observational cohort study with nested mixed methods case study (Trial registration: ISRCTN95551898). This subanalysis reports the results of quantitative cohort data obtained at baseline and up to 6 months post‐procedure. This study is reported with reference to the Strengthening the Reporting of Observational Studies in Epidemiology (STROBE) guidelines [10].

Thirty‐one UK sites recruited participants over a 46‐month period from May 2019 to March 2022. The study recruited patients who were 16 years of age or older with symptomatic PD. Patients who presented as an emergency with acute abscess were excluded.

Data were collected on a range of measures at several time points: baseline, day of procedure, Days 1 and 7 post‐procedure, 6 weeks post‐procedure and 6 months post‐procedure. Disease was defined using the International Pilonidal Sinus (IPS) classification as follows: type 1, only midline pit or sinuses; type 2, any midline disease with secondary sinus/es or abscess scar/s; type 3, any midline or secondary disease extending below the tip of coccyx; and type 4, any disease after treatment with definitive intent [11].

Patients were given a baseline questionnaire asking them to report on pain (rated on a scale of 0–10), health status (using the EQ‐5D‐5L tool) and impression of shared decision making (using the CollaboRATE measure) [12]. The CollaboRATE three‐question survey asks patients:

1. How much effort was made to help you understand your health issues?
2. How much effort was made to listen to what matters most to you about your health issues?
3. How much effort was made to include what matters most to you in choosing what to do next?

A mean score between 0 (indicating no effort was made) and 4 (every effort was made) was calculated.

The surgical procedure was reported by the performing clinician and broadly categorized into minimally invasive procedures [e.g. pit picking, Bascom I, glue, endoscopic pilonidal sinus treatment (EPSiT), laser, seton] or major skin excisional procedures with or without primary closure (e.g. Karydakis, Bascom cleft closure, rotational flap, midline closure, leave open with or without marsupialization) (Table 1).

**TABLE 1:** Breakdown of all procedures performed in the current study for treatment of pilonidal disease (*n* = 667) [14].

Procedure type	*n* (%)	Procedure category	*n* (%)	Procedure	*n* (%)
Major skin excision	397 (60)	Asymmetric closure	272 (41)	Bascom cleft **closure**	86 (13)
				Rotational Flap	22 (3)
				Karydakis	164 (25)
		Leave open	49 (7)	Leave open	43 (6)
				Leave open (marsupialization)	6 (1)
		Midline closure	76 (11)	Midline closure	76 (11)
Minimally invasive	270 (41)	Minimal excision	270 (41)	Bascom I	39 (6)
				EPSiT	44 (7)
				Glue	106 (16)
				Laser	11 (2)
				Pit picking	60 (9)
				Seton	10 (2)

Abbreviation: EPSiT, endoscopic pilonidal sinus treatment.

Post‐procedure outcomes were: pain (measured on Days 1 and 7, and at 6 weeks and 6 months); complications such as bleeding, dehiscence, discharge, seroma and infection (measured on Days 1 and 7, and at 6 weeks and 6 months); health status using the EQ‐5D‐5L (measured on Day 7 and at 6 weeks and 6 months); return to normal activities (measured on Day 7 and at 6 weeks and 6 months); length of time to healing and wound impact (measured at 6 weeks and 6 months); and decision regret (measured at 6 months). Decision regret was recorded using a five‐point scale, scored from 0 (low decision regret) to 100 (high decision regret) [13].

### Analysis

Analysis was performed using R, and descriptive statistics were used to report clinical outcomes with data presented as median with interquartile range (IQR) or number with a percentage to one decimal place as appropriate. Both univariate and multiple linear regression analyses were used to analyse the relationship between clinical outcomes and decision regret. For multiple linear regression analysis, iterative model development was undertaken, and the model was modified to optimize the Akaike information criterion (AIC) whilst maintaining representation of relevant, clinically plausible data. Data are presented as *β*‐coefficients with 95% CI.

### Ethical approval

Ethical approval was obtained from Cambridge South Research Ethics Committee (REC reference 18/EE/0370).

## RESULTS

From the 31 UK sites, 729 patients consented to be part of the cohort study. Participants were excluded from the analyses if they did not undergo a relevant procedure during the study (*n* = 45), if they were ineligible because of an incorrect diagnosis (*n* = 7) or if there was not enough information provided to categorize their procedure (*n* = 10). Six hundred and sixty‐seven participants were included in the cohort analysed, with follow‐up data at 6 months being available for 476 (71%). Seventy‐three per cent of participants were male, with an average age of 29 years.

### Operative outcomes

The breakdown of procedures performed have been published previously [14] and are detailed in Table 1. In short, 60% of patients received a major skin excisional procedure, most commonly asymmetric closure (40.8%), 15.1% had recurrent disease (IPS type 4). Participants with recurrent disease (defined as reporting any previous procedure excluding acute drainage) were more likely to undergo asymmetric closure than participants who did not have recurrent disease; by contrast, participants who did not have recurrent disease were more likely to recieve a minimally invasive procedure than participants with recurrent disease. Over half (56.0%) of participants with IPS type 1 underwent a minimally invasive procedure, whereas over half (53.4%) of participants with IPS type 4 underwent asymmetric closure.

Nearly half of participants experienced a complication during follow‐up (*n* = 301, 45.1%). Infection (26.2%, 175/667) and discharge (17.8%, 119/667) were the complications most commonly reported across all treatment approaches. Minimally invasive surgery resulted in a lower risk of complications [34.8% (94/270) vs. 52.1% (207/397)] and more rapid return to normal activities [87.6% (211/241) vs. 72.3% (270/373)] compared with patients who underwent a major procedure. This group also had the highest rates of wound healing at 6 months (74.6%). However, patients who underwent minimally invasive surgery were more likely to experience treatment failure at 6 months (27%) compared to patients who underwent a major procedure.

### Shared decision making

Overall participant satisfaction with their preoperative consultation was high; the median (IQR) of the CollaboRATE mean‐score response was 3 (3–4), meaning that participants felt ‘a lot of effort was made’ by the clinician to facilitate a shared decision. Participants who underwent a leave‐open procedure had the highest CollaboRATE mean‐score response, of 4 (3–4), and 43% of participants in this group gave a CollaboRATE top score, reflecting that ‘every effort’ was made to help the patient understand their health issue, listen to the things that matter most and include what matters most to the patient in choosing what to do next.

### Decision regret

Decision regret, measured at 6‐months post‐procedure, was low among patients in the cohort study [mean (SD) = 14.5 (16.7)] and was broadly similar across the procedure categories (Table 2). The majority (57.8%) of patients reported being either satisfied or very satisfied with their procedure. Participants who underwent a ‘leave open’ approach were most likely to be dissatisfied or very dissatisfied (14.3%); this approach was associated with the lowest rates of wound healing (59%) and return to normal activities (61.4%) across all treatment approaches. Participants who received ‘minimal excision’ reported low decision regret (53.0% were satisfied or very satisfied), and this approach was associated with the highest health utility and quality of life satisfaction at 6 weeks and 6 months.

**TABLE 2:** Preoperative shared decision‐making (evaluated using CollaboRATE) and post‐procedure outcome (decision regret, satisfaction with effect of treatment or care) patient‐reported scores, according to procedure category.

	Procedure category				
	Asymmetric closure	Leave open	Midline closure	Minimal excision	All
	(*n* = 272)	(*n* = 49)	(*n* = 76)	(*n* = 270)	(*n* = 667)
CollaboRATE mean score^a^					
*N* (%)	270 (99)	49 (100)	75 (99)	265 (98)	659 (99)
Median (IQR)	3 (3–4)	4 (3–4)	3 (3–4)	3 (3–4)	3 (3–4)
CollaboRATE top score given					
No, *N* (%)	182 (67)	28 (57)	51 (67)	155 (57)	416 (62)
Yes, *N* (%)	88 (32)	21 (43)	24 (32)	110 (41)	243 (36)
Decision regret scale^b^					
*N* (%)	198 (73)	35 (71)	51 (67)	173 (64)	457 (69)
Median (IQR)	10 (0–20)	8 (4–20)	8 (0–24)	8 (0–20)	8 (0–20)
Satisfaction with effect of treatment or care					
Very satisfied, *N* (%)	113 (42)	19 (39)	21 (28)	89 (33)	242 (36)
Satisfied, *N* (%)	61 (22)	9 (18)	18 (24)	54 (20)	142 (21)
Neither satisfied nor dissatisfied, *N* (%)	15 (6)	0 (0)	6 (8)	19 (7)	40 (6)
Dissatisfied, *N* (%)	3 (1)	6 (12)	6 (8)	9 (3)	24 (4)
Very dissatisfied, *N* (%)	9 (3)	1 (2)	0 (0)	6 (2)	16 (2)

^a^ Recorded at baseline. Scores ranged from 0 to 4: higher scores represent more perceived effort made by professional in the preoperative consultation.

^b^ Recorded at the 6‐month follow up. Scores ranged from 0 to 100: higher scores represent greater regret.

**FIGURE 1:**
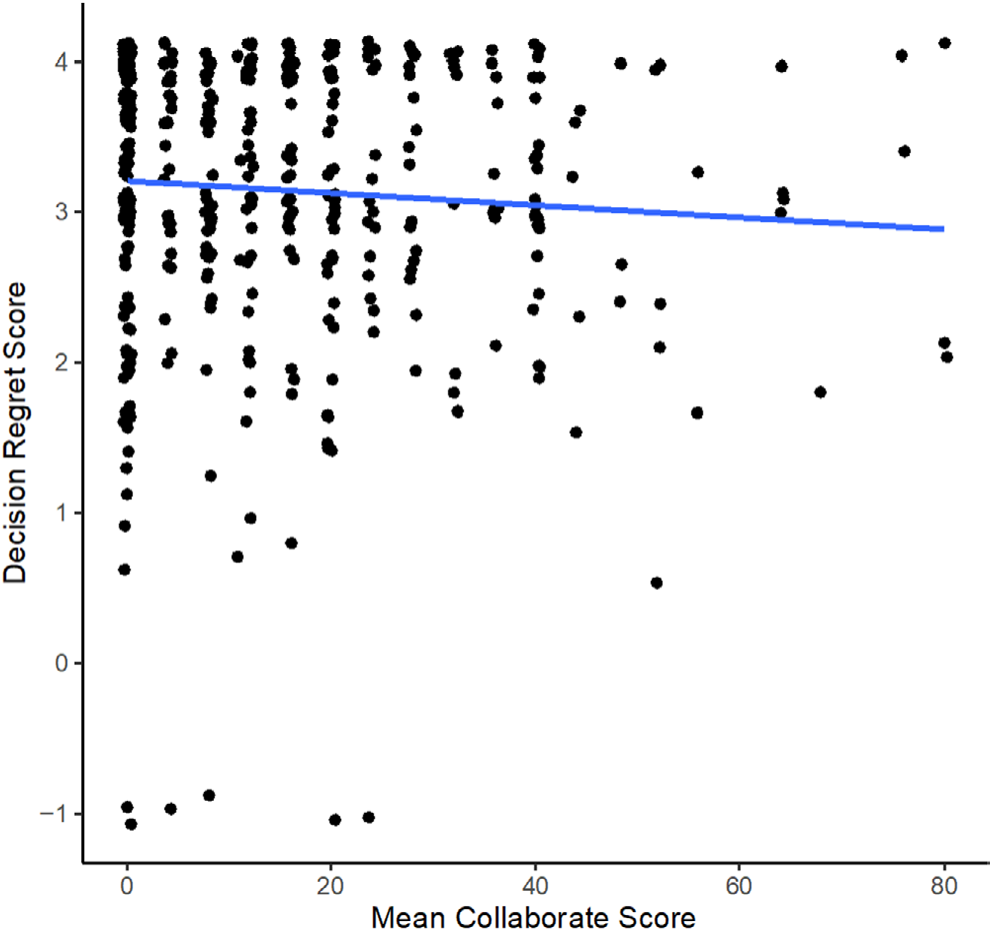
Scatterplot showing the relationship, in patients with pilonidal disease, between the CollaboRATE mean score at baseline and thes decision regret score at 6 months. Line represents fractional polinomial fit.

The relationship between the CollaboRATE mean score and decision regret is shown in Figure 1. The scores of most patients are located in the top left corner of the graph, indicating that, overall, participants were happy with their collaboration in treatment decision making and had few regrets regarding their procedure. Linear regression did not show an association between the CollaboRATE score and decision regret (*β* = 0.13; 95% CI: −12.99 to 7.01).

**FIGURE 2:**
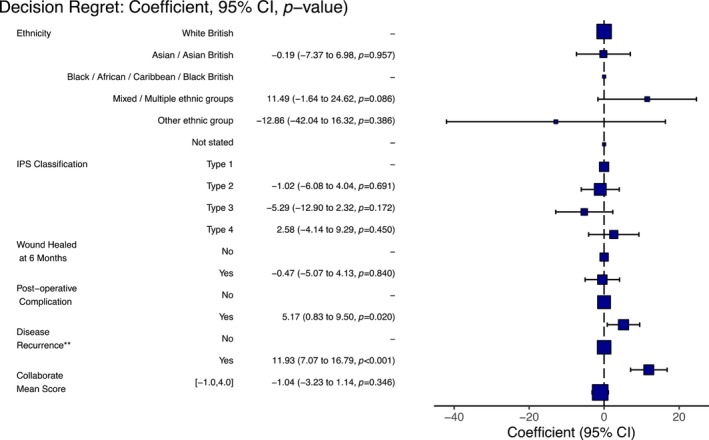
Multiple linear regression analysis of the association between clinical outcomes and decision regret. ** Recurrene ‐ patient required a further procedure or reported recurence.

The relationship between decision regret and clinical outcomes was analysed using both univariate and multiple linear regression analyses. In univariate analysis, the occurrence of postoperative complications (*β* = 3.21, 95% CI: −12.75 to 7.25, *p* < 0.001), prolonged wound healing (*β* = 4.04, 95% CI: –11.7 to 7.22, *p* < 0.001) and disease recurrence (*β* = 11.5, 95% CI: −10.6 to 9.4, *p* < 0.001) were all associated with decision regret. These clinical outcomes were further analysed in a multiple linear regression analysis (AIC =1582); both postoperative complications (*β* = 5.17, 95% CI: 0.83–9.50, *p* = 0.02) and disease recurrence (*β* = 11.93, 95% CI: 7.07–16.79, *p* < 0.001) remained significantly associated with decision regret (Figure 2).

## DISCUSSION

This paper reports the outcomes following operative management of Pilonidal Sinus Disease (PSD) and patient's assessment of their involvement in treatment decision preoperatively and decision regret postoperatively using data from one of the largest datasets of real‐world experience gathered on PSD to date. The data suggest that patients were happy with their level of involvement in shared decision making. The median CollaboRATE score, a tool for assessing the quality of shared decision making, was very high. In addition, 83% of those who were assessed for decision regret were either very satisfied or satisfied with the surgery.

The results are surprising given the fact that around one‐third of study participants had complications from surgery and in 25% the complications persisted for 6 months after surgery. However, decision regret was highest among patients who had undergone an excision and leave open procedure—this was associated with high levels of pain, wound management and extended healing time.

The low rates of decision regret within the cohort study are also surprising when we consider the results from a discrete choice experiment from the same study population [15]. Within this sub‐study, the two main treatment priorities for patients were reducing the risk of infection/persistence and shortening the time necessary to achieve recovery. The data suggest that patients are willing to accept less invasive procedures, such as glue or pit picking, which were associated with lower rates of complications and faster healing times but higher recurrence rates. In the ranking task, similar results were found: open surgery was ranked as the least favoured treatment option. This is understandable given the impact of prolonged wound‐care management on psychosocial wellbeing [16]. Despite this insight into patient priorities, most patients in the PITSTOP study underwent a major skin excisional procedure to treat their PSD and around one in 14 patients underwent an excision and leave open procedure.

One possible explanation for the inconsistency in favouring operations with a higher recovery burden is that surgeons may prioritize disease cure over symptom control and thus offer more invasive excisional approaches [4]. Patients can only make treatment decisions based on the information they are given and if minimally invasive procedures are not offered, this may be inadvertently compromising the shared decision‐making process. An incomplete range of treatment options being presented to patients may reflect the lack of training received by surgeons on the management of PD. The apprentice style of UK surgical training for PD means that surgeons may only be exposed to one or two techniques, which they are then likely to use throughout their career [4]. Consequently, shared decision making becomes difficult if a surgeon only specializes in one technique [17]. Clinical teams should ensure that patients are provided with sufficient information on the range of surgical procedures available, and manage their expectations regarding aftercare, complications and recurrence. In this study we did not categorize thesurgeon, with regards to operative experience or surgical techniques offered to patients, in the decision‐making dyad. Further modelling, taking into account surgeon skill set or operative volume, would be an interesting approach but unfortunately, we were not able to model for this in the present study.

Interestingly, the only clinical outcomes significantly associated with decision regret in both univariate and multivariate analyses were postoperative complications and disease recurrence. This presents a further challenge to both clinicians and patients when choosing between a minimally invasive or a major procedure. Opting for a major procedure may reduce the risk of disease recurrence; however, patients are more likely to experience prolonged wound healing and wound complications, with the reverse being true for minimally invasive procedures. There is also a trend towards increased decision regret in patients from the mixed/multiple ethnicities group. However, this was a small group of only eight patients and consequently it was difficult for this result to be meaningfully interpreted.

There are some concerns that self‐report measures of shared decision‐making may be inadequate and do not capture the quality of the interaction between patient and clinicians or the multi‐staged nature of the shared decision‐making process [18, 19]. Patient‐reported experience measures may be compromised by social desirability or acquiescence bias [20, 21], and more in‐depth open‐ended questions may reveal major problems from the same patients who report high levels of satisfaction on survey instruments [17, 22]. The self‐reported decisional regret from this study is consistent with the 1‐in‐7 rate reported across 73 surgical studies, in which regret was mainly associated with type of surgery, health outcomes and shared decision making [23]. The high level of satisfaction with preoperative discussions and the shared decision‐making process in this study may explain the low rates of decision regret. Postoperative outcomes are not the only factor valued by patients, as studies have consistently shown that when patients are involved in their treatment decisions, decision regret is lower, regardless of outcomes [23].

Several limitations need to be considered in relation to this study. Decision regret was evaluated 6 months post‐procedure; however, complete wound healing can take longer than 6 months to achieve and disease recurrence may occur over a period of many years post‐procedure. Therefore, the attitudes of participants, regarding decision regret, may be affected because of the short follow‐up period. Another limitation is the incompleteness of follow‐up data from the cohort study. Day 1 data were missing for 1 in 10 patients, and 6‐month data were only available for three‐quarters of patients. This is despite rigorous governance processes and dedicated research nurses arduously following up the patients. The incomplete data may reflect the demographic of the group, which tends to consist of young, active, working people, predominantly male. Such a demographic may be less likely to respond to follow‐up calls [24, 25].

## CONCLUSION

Despite significant rates of postoperative complications, treatment failure and protracted recovery after pilonidal sinus surgery, shared decision‐making appears to be carried out very well in the UK and decision regret among patients undergoing surgery for PSD is low. Nevertheless, it is likely that patients are not always offered the full remit of available treatment options.

## AUTHOR CONTRIBUTIONS

**J. Banks:** Writing – original draft; methodology; formal analysis; data curation; writing – review and editing. **Management Group PITSTOP:** Conceptualization; funding acquisition. **E. Lee:** Writing – review and editing; methodology; formal analysis; data curation. **M. J. Lee:** Conceptualization; funding acquisition; writing – review and editing; formal analysis; supervision. **S. R. Brown:** Conceptualization; funding acquisition; writing – review and editing; supervision.

## CONFLICT OF INTEREST STATEMENT

The authors have no conflict of interest to declare.

## Data Availability

The data that support the findings of this study are available from the corresponding author upon reasonable request.
